# Trends in the sizes and carbonaceous fractions of primary emitted particulate matter in China from 1960 to 2019

**DOI:** 10.1093/nsr/nwaf003

**Published:** 2025-01-16

**Authors:** Yuanzheng Zhang, Jin Li, Shuxiu Zheng, Rong Dai, Jinghang Wang, Yaqi Zhu, Wenxiao Zhang, Haoran Xu, Guofeng Shen, Huizhong Shen, Jianmin Ma, Xuejun Wang, Shu Tao

**Affiliations:** Institute of Carbon Neutrality, Laboratory for Earth Surface Processes, College of Urban and Environmental Sciences, Peking University, Beijing 100871, China; Institute of Carbon Neutrality, Laboratory for Earth Surface Processes, College of Urban and Environmental Sciences, Peking University, Beijing 100871, China; Institute of Carbon Neutrality, Laboratory for Earth Surface Processes, College of Urban and Environmental Sciences, Peking University, Beijing 100871, China; Institute of Carbon Neutrality, Laboratory for Earth Surface Processes, College of Urban and Environmental Sciences, Peking University, Beijing 100871, China; Institute of Carbon Neutrality, Laboratory for Earth Surface Processes, College of Urban and Environmental Sciences, Peking University, Beijing 100871, China; Institute of Carbon Neutrality, Laboratory for Earth Surface Processes, College of Urban and Environmental Sciences, Peking University, Beijing 100871, China; Institute of Carbon Neutrality, Laboratory for Earth Surface Processes, College of Urban and Environmental Sciences, Peking University, Beijing 100871, China; Institute of Carbon Neutrality, Laboratory for Earth Surface Processes, College of Urban and Environmental Sciences, Peking University, Beijing 100871, China; Institute of Carbon Neutrality, Laboratory for Earth Surface Processes, College of Urban and Environmental Sciences, Peking University, Beijing 100871, China; School of Environmental Science and Engineering, Southern University of Science and Technology, Shenzhen 518055, China; Institute of Carbon Neutrality, Laboratory for Earth Surface Processes, College of Urban and Environmental Sciences, Peking University, Beijing 100871, China; Institute of Carbon Neutrality, Laboratory for Earth Surface Processes, College of Urban and Environmental Sciences, Peking University, Beijing 100871, China; Institute of Carbon Neutrality, Laboratory for Earth Surface Processes, College of Urban and Environmental Sciences, Peking University, Beijing 100871, China; School of Environmental Science and Engineering, Southern University of Science and Technology, Shenzhen 518055, China

**Keywords:** primary particulate matter, size, carbonaceous PM, temporal trend

## Abstract

The health impacts of particulate matter (PM) depend on its concentration, size and composition. Herein, we quantified the changes in the emissions of primary PM_2.5_, PM_2.5–10_ and PM_>10_ with aerodynamic diameters of <2.5 μm, 2.5–10 μm and >10 μm, respectively, black carbon (BC), and organic carbon (OC) to address the changes and driving factors. The temporal trends of PM emissions follow Kuznets curves, with 1995 as the peak year when the gross domestic product per capita was only US$1023, showing a late-mover advantage. The fractions of PM_2.5_ : PM_2.5–10_ : PM_>10_ and BC : OC : non-carbonaceous-PM_2.5_ from various sectors varied following different trajectories. The mass fractions of PM_2.5_ : PM_2.5–10_ : PM_>10_ from iron–steel production industries changed from 21% : 12% : 67% in 1960 to 50% : 13% : 37% in 2019, showing a decrease in PM size. The fractions of BC were linearly correlated with PM_2.5_, whereas the dependence of OC on PM_2.5_ differed before and after 1995, owing to changes in residential emissions. Various factors influencing the changes in size and carbonaceous fraction were explored. The major factors were the promotion of dust-removal capacity and the transition in residential energy from solid fuels to emission-free fuels, which increased the fractions of fine PM and carbonaceous fraction.

## Teaser

While total particulate matter emissions decreased in recent years, finer size fractions and carbonaceous fractions increased.

## INTRODUCTION

China has emerged as the leading manufacturer of various commodities globally over the past several decades [1]. Rapid economic development and industrialization also deteriorate the environment, which poses a severe threat to the nation [2]. Air pollution represents a major threat due to its wide-ranging adverse health outcomes [3]. Although air quality has improved substantially during the past decade [4], 1.85 million premature deaths occurred because of air pollution in China in 2019 [5].

Particulate matter (PM), an air pollutant, is primarily emitted or secondarily formed from various precursors [6]. Although the contribution of secondary aerosols is increasing, primary PM is important [6,7]. The health impacts of atmospheric PM are quantified based on the exposure of the population to size- and component-dependent PM_2.5_ (aerodynamic diameter <2.5 μm) [8]. PM finer than PM_2.5_ can penetrate deep into the lungs [9,10], causing more damage than PM_2.5_ [11]. Moreover, the adverse health effects of airborne black carbon (BC) and organic carbon (OC) are more potent than those of PM_10_ (aerodynamic diameter <10 μm) at the same concentration levels [12–15].

Over the past few decades, PM emissions and their influence on health and actions to abate air pollution in China were extensively studied, and emission control technologies were installed widely in industries [16]. The efficiency of dust-removal facilities depends on the size and composition of PM [17–19]. The long-term evolution of size-segmented and carbonaceous PM emissions is yet to be studied. Factors such as the transition in industrial structures and energy, the phasing of traditional processes and technologies, and the upgrading of mitigation measures responsible for this evolution need to be quantified to understand the relationship between air pollution control and health benefits. The re-evaluation of these factors can modify control strategies and enhance effectiveness. Thus, the general trend of PM emissions based on their size, carbonaceous fraction and total emissions should be investigated.

This study aimed to quantify temporal trends in the size and carbonaceous fraction of primary PM emissions from 1960 to 2019. Recently updated emission inventories were used to provide the necessary data for quantifying emissions of three PM size fractions [PM_2.5_, PM_2.5–10_ (aerodynamic diameter between 2.5 and 10 μm) and PM_>10_ (aerodynamic diameter >10 μm)], and BC and OC for this study (gems.pku.edu.cn; gems.sustech.edu.cn) (see Methods). The size and composition dependence on the sources were analyzed based on the quantified trajectory of size and carbonaceous fractions of primary PM. Significant factors were distinguished to quantify their contributions to these changes.

## RESULTS AND DISCUSSION

### Variation in size and composition of primary PM

Over the past several decades, PM concentrations in the ambient air in China's mainland increased until 2014, followed by a rapid decrease [20]. The overall trend was positively and negatively attributed to population growth, urbanization, industrialization [21], industrial process upgrading [22], residential energy transition [23] and increases in end-of-the-pipe abatement efforts [24]. Apart from secondary aerosols, primary PM from direct emission sources is always a vital PM component in ambient air [6,25].

**Figure 1.**
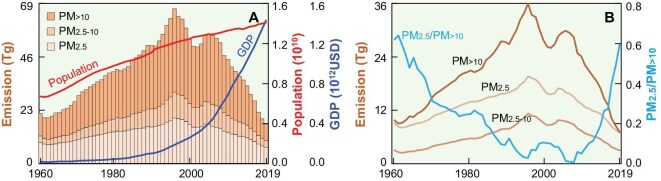
Temporal emission trends of PM_>10_, PM_2.5–10_ and PM_2.5_ from 1960 to 2019. (A) Overlay area charts of the three fractions compared with the population and GDP. (B) Line charts for the emissions of PM_>10_, PM_2.5–10_ and PM_2.5_ together with PM_2.5_/PM_>10_ ratios.

Temporal trends in emissions of three primary PM sizes of PM_>10_, PM_2.5–10_ and PM_2.5_ from 1960 to 2019 are shown in Fig. 1A as a stacked area chart. The changes in the gross domestic product (GDP) and total population, as indicators of socioeconomic development, are presented as the lower and upper solid lines in Fig. 1A. The emissions of PM_>10_, PM_2.5–10_ and PM_2.5_ increased gradually before 1995, then fluctuated and decreased after 1995 rapidly. The decline in PM emissions after 1995 was in opposition to the steady increase in population and GDP, demonstrating successful emission abatement during the last three decades [16]. The bell-shaped trends of annual emissions of primary PM are consistent with the environmental Kuznets curve, supporting the hypothesized two-stage dependence of air quality on economic development [24]. The positive factors affecting emissions have predominated the general trend of air quality degradation since 1960. At the same time, a series of abatement efforts were the leading factors in reducing PM emissions after 1995. Although ambient air pollution control campaigns (mitigation actions) were not launched until 2013 [26], efforts to reduce air pollution started earlier [16]. Dust-removal facilities were installed in power stations and industries in the mid-1990s [16], and beehive coke ovens were banned during the same period [27]. Residential energy sources have switched from solid fuels to low-emission fuels in the past four decades [23,28]. Thus, the abatement efforts offset, to some extent, the increased emissions since 1995. The GDP per capita of PM emissions was US$1023 in 1995 (purchasing power parity to 2019). The GDP per capita at the turning point of the Kuznets curve was more than an order of magnitude lower than those in developed countries such as Germany (US$18 192 cap^−1^), the United Kingdom (US$14 419 cap^−1^), Japan (US$23 014 cap^−1^) and the United States of America (US$41 115 cap^−1^), showing remarkable late-mover advantage [24,29].

Although the general emission trends of PM_>10_, PM_2.5–10_ and PM_2.5_ are similar, the differences can still be identified, and are illustrated as solid brown lines for individual sizes in Fig. 1B. Annual emissions before 1995 increased 1.2- (PM_2.5_), 2.8- (PM_2.5–10_) and 2.7-fold (PM_>10_) from 1995 baselines, indicating a rapid increase in the emissions of coarse PM. The emissions of coarse PM decreased rapidly after 1995, indicating that the change in coarse PM dominated the total PM variations. The annual emissions of PM_>10_ in 2019 were 20% of their peak value in 1995, 26% and 35% lower than those of PM_2.5–10_ and PM_2.5_, suggesting differences in the effectiveness of abatement measures of PM with different sizes. The PM_2.5_/PM_>10_ ratios decreased from 0.71 in 1960 to 0.40 in 2007, and increased to 0.70 again in 2019. The trend of the ratio highlighted by the blue solid line further confirms that fine PM mitigation requires more targeted control measures than coarse PM. The reasons for these changes are elaborated in the following section in detail.

**Figure 2.**
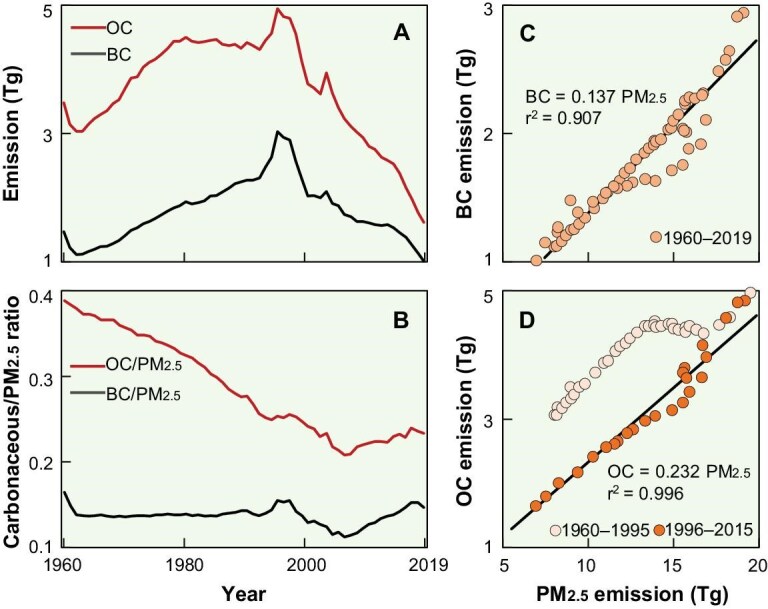
Temporal trends of annual emissions of (A) BC and OC, and (B) BC/PM_2.5_ and OC/PM_2.5_ from 1960 to 2019. The dependence of the annual emissions of (C) BC and (D) OC on PM_2.5_.

The compositions of primary PM from various sources altered over the period, resulting in changes in particle size fractions. The annual emissions of BC and OC from 1960 to 2019 are shown in Fig. 2A, displaying increasing trends before 1995 followed by a rapid decrease after 1995. BC and OC, as incomplete combustion products, exhibited similar trends to those of PM. Differences between BC and OC aerosols can be discerned via the temporal variations of BC/PM_2.5_ and OC/PM_2.5_ ratios for 60 years (Fig. 2B). The ratios were calculated based on PM_2.5_ because almost all BC and OC from primary combustion comprised fine PM [30,31]. Figure 2B shows that OC/PM_2.5_ ratios decreased from 0.38 in 1960 to 0.21 in 2006 and increased slightly to 0.23 by 2019. In comparison, BC/PM_2.5_ ratios did not change much prior to 1995, and followed a similar trend as OC/PM_2.5_ ratios after. The differences were due to changes in emissions from the residential sector, coke production and brick production. The relative constant BC/PM_2.5_ ratios suggest that BC in PM_2.5_ changed little, which was confirmed by a significant linear correlation (*p* < 0.01) (Fig. 2C). Alternately, although the annual emissions of OC and PM_2.5_ were also significantly correlated (*p* < 0.05), the relation deviated from linearity before 1995 (Fig. 2D). The difference in OC/PM_2.5_ ratios before and after 1995 is mainly attributed to the change in source compositions. The emission sources were residential biomass combustion (25%–61%) before 1995. The average emission factor (EF) of OC from residential sources was 2.82 ± 1.88 g kg^−1^, more significant than the mean EF of 0.25 ± 0.18 g kg^−1^ from other sources [21,32,33]. Therefore, dominant residential emissions before 1995 notably deviated OC/PM_2.5_ ratios from the overall linear relationship (Fig. 2D).

The emissions of PM_>10_, PM_2.5–10_, PM_2.5_, BC and OC also varied in space with temporal changes. Figure S1A and B show that the annual emissions of PM_2.5_ and PM_>10_ in 2019 in eastern China were higher than in other regions. The major PM_>10_ emission sources were identified in Henan and most large cities with intensive brick, lime and cement industries [34–36]. The PM emission densities were distributed relatively evenly in the space, partially owing to strong emissions from discrete rural households using solid fuels [37]. The geographical distributions of the annual emissions of BC and OC in 2019 are shown in Fig. S1C and D, indicating similar patterns to that of PM_2.5_. Although emissions in 2019 were mapped and total emissions changed substantially in the past, the general spatial distribution patterns of PM, OC and BC emission densities did not change notably over these years.

### Temporal variations in source profiles

**Figure 3.**
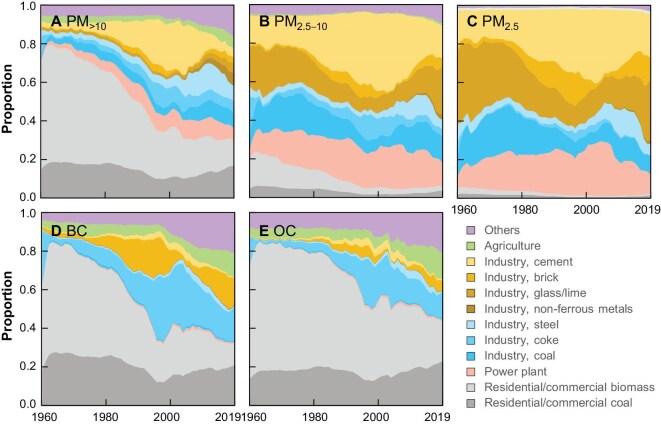
Relative contributions of major sources to annual emissions of (A) PM_>10_, (B) PM_2.5–10_, (C) PM_2.5_, (D) BC and (E) OC from 1960 to 2019. In addition to those listed explicitly, all other sources are summed up as ‘others’.

Major reasons for the variations in quantities, sizes and BC/OC compositions of primary PM were attributed to different sources and their emission intensities. Figure 3 shows the relative contributions of primary sources to the total emissions of PM_>10_, PM_2.5–10_, PM_2.5_, BC and OC from 1960 to 2019. Among 146 emissions sources compiled in the updated inventories (gems.pku.edu.cn; gems.sustech.edu.cn), 11 major source types are shown individually, whereas the others are marked together as ‘others’. Figure 3A shows that the sources of PM_2.5_ were dominated by residential/commercial solid fuel burning in the early years, which is different from those of PM_>10_ and PM_2.5–10_ (Fig. 3B and C). The relative contributions of residential solid fuel emissions over the six decades were reduced from 71.5% to 29.7% (PM_2.5_), 19.7% to 6.2% (PM_2.5–10_) and 5.3% to 2.1% (PM_>10_). The dominating residential source of PM_2.5_ was gradually replaced by other sources such as industrial coal consumption, power generation and the production of iron and steel, cement, glass and lime [1]. Similar sources of PM_2.5–10_ and PM_>10_ were from coal-fired power plants and various industrial processes (cement, glass and lime production industries). Apart from the changes in source profiles, another important cause affecting the change in PM size after 1995 was an increase in dust-removal capacities in power stations, industries and motor vehicles [16]. The penetration rates of dust-removal facilities in coal-fired power stations increased from 86.5% in 1995 to 95.6% in 2005 [17]. The end-of-pipe abatement facilities removed coarser PM more efficiently than finer PM [18], leading to a sharper decline in PM_>10_ emissions than PM_2.5_ emissions and a shift in size fractions after 1995. For example, fine PM (10%) and coarse PM (70%) were removed using cyclones in the cement industry [38]. The average PM_2.5_ removal efficiency of precipitators is 97.1%, <99.0% of PM_2.5–10_ [18,34].

Figure 3D shows that the temporal trend of BC sources is similar to that of PM_2.5_ except for strong contributions from the coke and brick production industries and low contributions from cement and iron–steel industries after the 1970s. The temporal trend of source contributions to OC emissions is similar to that of BC except for the slow decline in residential and commercial contributions (Fig. 3E). Residential and commercial sources contributed 43.5% to total OC emissions in 2019 but 32.8% to BC [21]. The relation between OC and PM_2.5_ before 1990 deviated significantly from a simple linear relation to a rapid decline in the relative contribution of biomass fuel consumption in residential sectors.

**Figure 4.**
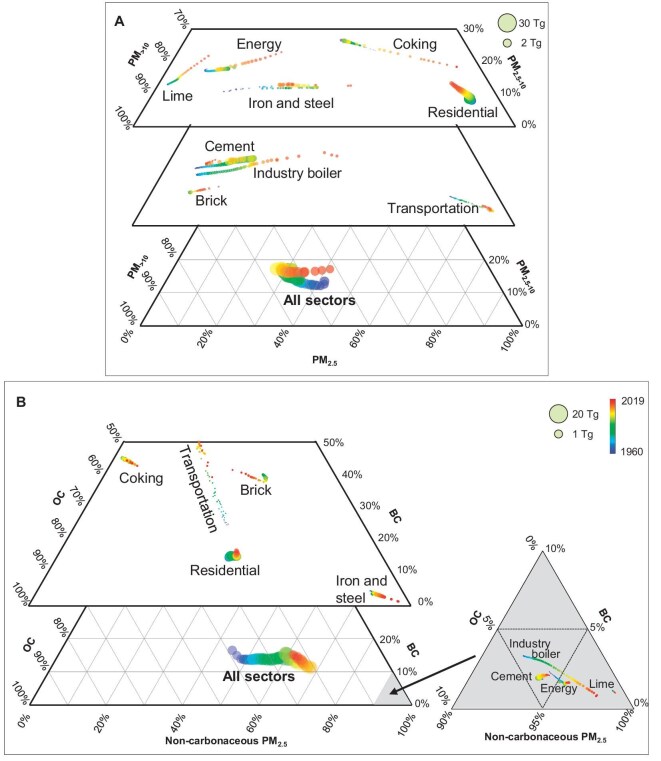
Trajectories of (A) PM_2.5_ : PM_2.5–10_ : PM_>10_ and (B) BC : OC : non-carbonaceous-PM_2.5_ fractions from 1960 to 2019. Total emissions and emissions from nine primary sources are shown. Symbol sizes are proportional to annual emissions, whereas colors represent emission years.

Temporal trends in the changes in size distribution of PM from various sources differ remarkably. Figure 4A shows the differences as fractions of PM_2.5_ : PM_2.5–10_ : PM_>10_ in ternary charts. The three axes represent the relative contributions of PM_2.5_ (bottom), PM_2.5–10_ (right) and PM_>10_ (left). As PM_2.5–10_ contributed no more than 30% in all cases, only part of the charts with PM_2.5–10_ <30% are shown. The contribution of PM_2.5–10_ emissions from all sectors varied slightly, consistently remaining between 10% and 20%. The trajectories of individual sources differ remarkably. The mass fractions of PM_2.5_ : PM_2.5–10_ : PM_>10_ from iron–steel production varied from 21% : 12% : 67% in 1960 to 50% : 13% : 37% in 2019, indicating a change in size fraction. Fine PM dominates the residential and transportation sectors at the bottom-right corners. The PM size associated with the transportation sector was smaller than that of most other sources. 78.5% of PM_2.5_ emission was released from the transportation sector in 1960, and the coarser PM was further reduced to 88.6% in 2019 because fine PM is more challenging to remove via tail dust-removal facilities [39]. An increase in coal/biomass fuel ratios and refuse incineration shifted the trajectory toward coarse PM in the residential sector [23,40]. Industrial activities such as cement, brick and lime production industries and power stations emitted >70% of PM_>10_ [19,34,41–44]. The PM emissions from most sources have shifted to fine sizes because fine particles are more difficult to remove via end-of-pipe facilities [18]. Affected by the different and sometimes opposite moving directions of the trajectories of individual sources, the fractions of PM_2.5_ : PM_2.5–10_ : PM_>10_ in total emissions changed from 41% : 14% : 45% in 1960 to 31% : 15% : 54% in 1988, and 41% : 17% : 42% in 2019, showing a reversed and nearly closed trajectory. This trend will continue as the general directions of process updating, energy transition and abatement technology will remain the same.

Similar to PM size fractions, primary PM's BC and OC contents also varied over time. BC and OC comprise a major fraction of PM_2.5_. Hence, the relation between BC, OC and other compositions of PM_2.5_ (non-carbonaceous PM_2.5_) was quantified and is presented in ternary charts in Fig. 4B. Trapezoid charts were partially drawn because BC content was <50% in all cases. The fraction of BC : OC : non-carbonaceous-PM_2.5_ is shown for emissions from major sectors and all sources from 1960 to 2019. BC and non-carbonaceous PM_2.5_ increased from 61.2% to 76.9%, and OC decreased from 38.8% to 23.1%, whereas BC varied slightly between 11.2% and 16.4% during this period. The overall decarbonizing trend was driven by changes in emissions from industry boilers and productions of energy, iron-and-steel, coke, lime and cement. Meanwhile, the trajectories of transportation and brick production went in the opposite direction. For transportation emissions, increased BC fractions were associated with an increase in diesel-fueled vehicles. In comparison, the BC fractions changed slightly over the period and BC : OC : non-carbonaceous-PM_2.5_ fractions from residential emissions varied much less than those from PM_2.5_ : PM_2.5–10_ : PM_>10_ fractions.

### Factors driving change in PM sizes

**Figure 5.**
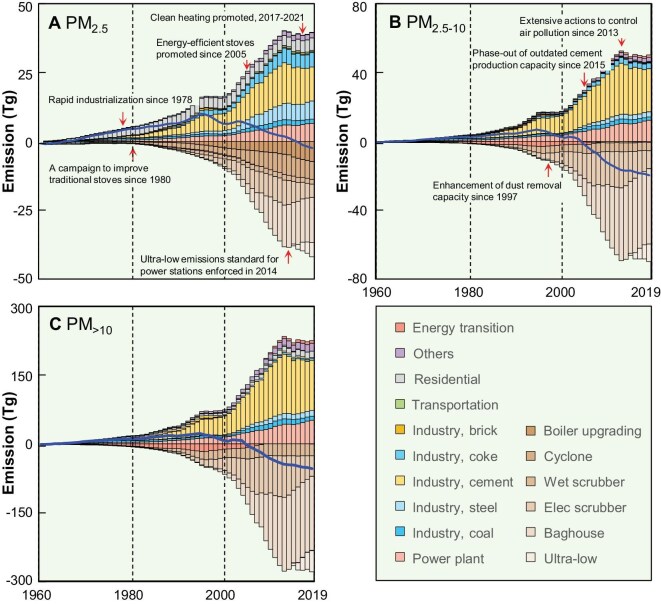
Decomposition of driving factors affecting accumulative emissions of (A) PM_2.5_, (B) PM_2.5–10_ and (C) PM_>10_ from 1960 to 2019, with 1960 as the baseline. The net effects of positive and negative drivers are shown as solid lines. Significant mitigation actions promoted in China during this period are marked.

Changes in PM emissions were caused by various factors. The different effects of these factors on PM size fractions changed PM size fractions in their emission evolution. The major factors impacting the emissions of PM_>10_, PM_2.5–10_ and PM_2.5_ accumulated over 60 y with 1960 as a baseline are shown in Fig. 5. The major drivers were population growth, urbanization-associated changes in energy consumption, residential energy switch, the increased production of cement and other products, the installation of exhaust gas treatment apparatus promoted by tightened emission regulations, the enhancement of end-of-pipe dust-removal capacity for power stations and industries, etc. [4,45,46,21]. The magnitudes of actual emission changes (blue solid lines, Fig. 5) were smaller than most drivers because positive and negative driving factors largely balance each other. Abatement measures as negative drivers were effective because total emissions would be extremely high without mitigation actions. The emissions of PM_2.5_ increased by 10.0 Tg from 1960 to 1995. The emissions would have reached 48.7 Tg by 2019 without negative drivers such as dust-removal facilities, improved processes and energy transition. However, accumulative adverse effects in 2019 maintained the emissions at 6.8 Tg, reducing the emissions by 41.9 Tg. The drivers of PM_2.5–10_ and PM_>10_ were similar due to their similar sources, whereas those of PM_2.5_ were different. Thus, the two fractions of PM_2.5–10_ and PM_>10_ were combined and termed PM_>2.5_. Cement production is the most critical driver, rapidly increasing the emissions of PM_>10_, PM_2.5–10_ and PM_2.5_, respectively. Coal-fired power plants are crucial for all size fractions. Industrial, coke production and residential emissions contributed primarily to the changes in PM_2.5_ emissions. The reduced emissions were mainly attributable to installing various end-of-pipe dust-removal facilities. Other negative drivers include residential energy transition and the upgradation of industrial boilers [45,47]. Various mitigation actions promoted the drivers during these years. The extensive promotion of dust-removal equipment in power stations and industries since 1997 has reduced emissions of PM_>10_, PM_2.5–10_ and PM_2.5_, particularly coarse PM [16]. Similarly, the phasing out of beehive coke ovens [27] and subscale cement kilns [44] from 1996 and 2015 rapidly reduced emissions from these sources. The ultralow emission control campaign in coal-fired power plants promoted since 2014 became a new and important negative driver affecting PM_>10_, PM_2.5–10_ and PM_2.5_ [48,49].

**Figure 6.**
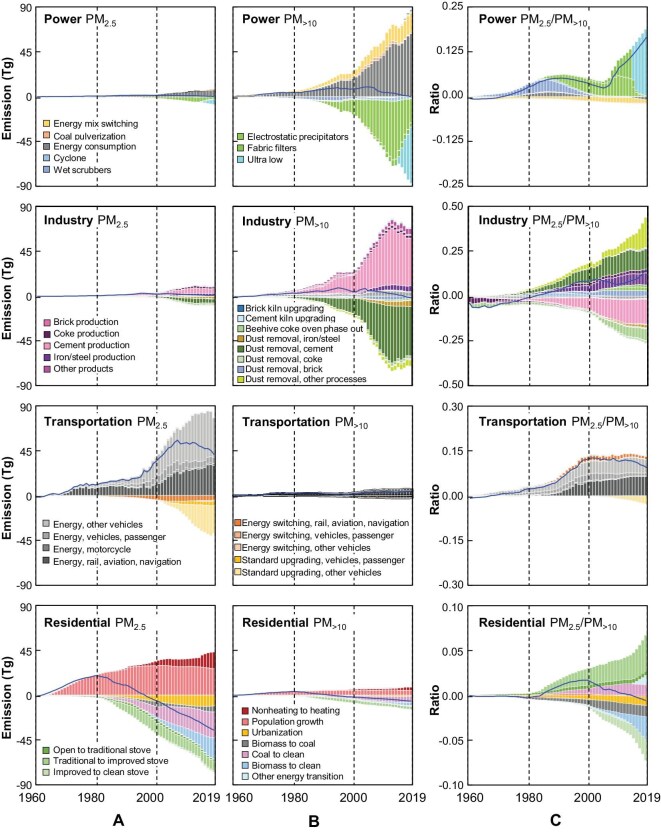
Decomposition of driving factors affecting accumulative emissions of PM_2.5_ (Column A), PM_>10_ (Column B) and PM_2.5_/PM_>10_ (Column C) for four sectors (power generation, industry, transportation and residential) from 1960 to 2019. The driver PM_2.5_/PM_10_ emission ratios were derived as ratios of quantified drivers for PM_2.5_ and PM_>2.5_.

The factors driving the changes in PM size fractions were investigated. The drivers affecting the changes in PM_2.5_, PM_>2.5_ and PM_2.5_/PM_>2.5_ ratios accumulatively from 1960 to 2019 were compared in electricity production, industry (both industrial boilers and industrial processes), transportation and residential sectors (Fig. 6). The solid blue lines presented in Fig. 6 show the net difference between the accumulative positive and negative effects. The four sectors can be divided into two categories based on the changes in PM_2.5_ and PM_>2.5_ emissions: fire power generation/industry and transportation/residential. The increasing emissions of primary PM from energy production and industrial sectors revealed that PM_>2.5_ was approximately an order of magnitude higher than PM_2.5_. Increasing electricity and cement production industries are major factors driving the increased emissions of PM_>2.5_. Coal pulverization increased coarse PM emissions from power stations [45]. By contrast, various dust-removal technologies increased the fraction of PM_2.5_ emissions. The average efficiency of dust-removal facilities for removing PM_>10_ varied from 90.0% to 99.99%, whereas the average efficiency of cyclones for removing PM_2.5_ is low (15%) [18]. Cyclones, electrostatic precipitators, wet scrubbers, fabric filters and ultrafine control were generally promoted consecutively from 1970 with increased efficiency and cost. Various positive or negative drivers acted synchronously during the entire period in the industrial sector. The strong influence of ultralow emission technology on rapidly increasing fine PM ratios in the power sector can be discerned. Similarly, increased fuel consumption in the transportation sector enhanced the emissions of fine PM from the mid-1990s to the mid-2010s.

The primary drivers affecting accumulative changes in PM emissions from transportation and residential sectors act strongly on fine PM. The increase in energy consumption in the transportation sector and population growth in the residential sector are positive drivers. The effects of rapidly increasing fuel consumption in the transportation sector, particularly diesel for heavy-duty motor vehicles and trains, could not be balanced by negative factors such as constrained emission standards. With the shift from coal to gasoline or electricity for fueling trains, these drivers reduced the size of PM emissions. The population growth and increase in household heating systems (electric heaters) increased emissions in the residential sector. Traditionally, the areas south of the Qin Mountains and Huai River did not use household heating systems, although the daily mean temperature is occasionally <0°C in winter [50]. However, heating system use has increased during winter owing to improved living conditions [37], which increased PM_2.5_ emissions in these areas.

Urbanization and the transition of residential cooking/heating energy and stoves are negative drivers, which have reduced emissions since the 1980s. The migration of people from rural areas to cities under urbanization substantially reduced biomass fuel consumption [51], a critical factor in the transition from solid fuels to clean energy for cooking [28,52]. A clean heating campaign implemented in the North China Plain from 2013 to 2017 under government intervention has facilitated a rapid switch of heating energy from coal to natural gas or electricity [53]. Thus, most negative drivers overwhelmed the positive drivers, and this was characterized by a continuous decrease in PM_2.5_ emissions. Several drivers affected PM size fractions oppositely. Upgrading residential stoves and shifting coal to clean energy in the residential sector decreased the PM size fractions.

Since BC and OC are strongly associated with PM_2.5_, the drivers contributing to their emission changes are similar to those for PM_2.5_ and are hence not discussed here.

### Limitations and implications

To compile the global inventories for more than 100 sources in seven sectors at high spatial resolution, detailed data are needed for activities and EFs. Although efforts have been made to gather as many data as possible, from the literature to field surveys, high uncertainty is unavoidable, which is a major limitation of the study. Monte Carlo simulation was used (see Methods) throughout the compilation procedure to quantify uncertainties in activities, EFs, size fractions and end-of-pipe control rates (see Table S2). To reduce uncertainty in spatial disaggregation, sub-national data were collected for large countries including China, India, the USA, Canada, Australia, Brazil, South Africa, Indonesia, Turkey, Russia, Mongolia and 13 other developing countries. Within these sub-national regions, spatial proxies were applied primarily due to data gaps. In fact, many data, especially those for developing countries, do not even exist. Relatively high uncertainty can be seen in Fig. S2.

Over the past several decades, extensive efforts have been made in China to abate air pollution. The rapid reduction in primary PM emissions and improvement in air quality indicates the efficiency of the emission control strategies and efforts [2]. Although emission reduction started 3 decades ago, remarkable improvements in air quality could only be observed 10 years ago. The growing emissions were finally offset by mitigation efforts, and peak emissions were achieved earlier than expected. Otherwise, the emissions would have been high in the absence of these efforts due to the rapid expansion of emission activities.

The results from the present study revealed that total PM emissions decreased significantly. However, the fraction of fine PM increased, primarily because of the shifts in sources and different efficiencies of various dust-removal technologies. Given that a majority of OC and BC are associated with fine PM_2.5_, BC and OC proportions, primarily in emitted PM, also increased. Considering that the health impact assessment is based on PM_2.5_, changes in PM size fractions and carbonaceous fractions will create biases and likely be underestimated in risk evaluation [14,15], which needs to be quantitatively addressed in the future based on the size and composition. Specifically, the toxicity and epidemiology of ultrafine and carbonaceous particles should be quantified and studied.

Although air quality in China has improved substantially in recent decades [54,55], future improvement is still challenging [2] because the emissions from coarse PM sources that can be eliminated easily have already been reduced substantially. However, fine PM sources are costly to remove, resulting in a higher proportion of PM total emission [5,56].

It is worthwhile to note that the present study solely focused on primary PM. The changes in the size fraction and composition of secondary aerosols need to be addressed in the future.

## METHODS

### Emission inventory compilation

Emission inventories of total suspended particulates PM_10_, PM_2.5_, BC and OC were compiled using a bottom-up approach based on emission activities, technology categories, EFs and end-of-pipe dust-removal capacities (gems.pku.edu.cn; gems.sustech.edu.cn). The quantified emissions of these pollutants from 146 sources were classified into eight sectors: power generation, industrial energy consumption, industrial process, transportation, agricultural, residential, commercial and natural (Table S1). The inventories were rooted in the Peking University inventories (inventory.pku.edu.cn) with substantial updating (Table S1). The emissions were spatially (0.1° × 0.1°) and temporally (monthly) resolved [34,57,22]. Although natural sources were included, 98% of the total emissions were dominated by anthropogenic contribution. Hence, natural emissions are neglected in this study.

### Data sources

Four databases comprising activities, technology categories, EFs and end-of-pipe dust-removal penetration rates were compiled. Among 50 air pollutants covered by the inventories, three PM size fractions (PM_>10_, PM_2.5–10_ and PM_2.5_) and two carbonaceous PMs (BC and OC) were considered in this study. Activity data included the consumption of various energy types (coal, oil, gas, biomass and waste) in six anthropogenic sectors and the production of major industrial products. The energy data were collected mainly from the International Energy Agency. The data from the residential sector were obtained from two rounds of nationwide surveys for rural energy consumption [23,37] and various Chinese Statistical Yearbooks for the urban population [58]. Industrial production data were collected from the World Steel Association, the US Geological Survey and several previous studies [59,27]. EFs were gathered from a thorough literature review of those measured for more than 2000 field-collected samples in our laboratory. After categorization and cleanup, an EF database with >8000 records was adopted. The penetration rates of various end-of-pipe dust-removal technologies such as cyclones, electrostatic precipitators, wet scrubbers and fabric filters were obtained from previous studies [18,19,34].

### Driver quantification

The reduction in primary PM emissions was attributed to various causes, which were quantified as accumulative effects on emission changes. The identified factors included changes in production and energy consumption, upgradation of industrial processes such as different types of cement kilns and coke ovens, dissemination of coal pulverization, dust-removal technologies such as cyclones, electrostatic precipitators, wet scrubbers and fabric filters, enhanced exhaust control technologies for motor vehicles, and residential energy transition owing to population growth, urbanization, living condition improvement and government intervention. These drivers played vital roles, positively or negatively, in PM emissions and emission evolution. Structural decomposition analysis was applied to quantify the effects of various drivers using 1960 as a base year [21,22], and the results are presented accumulatively from 1960 to 2019. The ratios of quantified drivers for PM_2.5_ and PM_>2.5_ (sum of PM_2.5–10_ and PM_>10_) were calculated to directly address the factors influencing the change in size-resolved PMs.

### Uncertainty analysis

The overall uncertainty associated with activity strengths, EFs and the penetration rates of end-of-pipe abatement technologies was characterized via a 10 000-fold Monte Carlo simulation. Uniform distributions were assumed for activity data with 10% or 5% of coefficients of variation (CV) for residential biomass fuel consumption or all other sources based on databases compiled previously (gems.pku.edu.cn; gems.sustech.edu.cn). EFs were considered to be log-normally distributed with CV based on the EF database. The penetration rates of various abatement technologies were assumed to be normally distributed with 5% of CV. The uncertainty analysis results were presented via intervals from the 25th to the 75th percentiles. Excel 2021 and MATLAB R2021a were used for data analysis, and ArcGIS 10.6 was used for mapping.

## Supplementary Material

nwaf003_Supplemental_Files
