# Supplementary material for: Trends in the sizes and carbonaceous fractions of primary emitted particulate matter in China from 1960 to 2019
Source: Natl Sci Rev. 2025 Jan 16;12(3):nwaf003. doi: 10.1093/nsr/nwaf003 (PMC11831801; doi:10.1093/nsr/nwaf003)
Supplement: nwaf003_Supplemental_Files [file nwaf003_supplemental_files.zip › SI for NSR_nwaf003.docx]

**Supporting Information for**

**Trends in the sizes and carbonaceous fractions of primary emitted particulate matter in China from 1960 to 2019**

Zhang, Yuanzheng^1^; Li, Jin^1^; Zheng, Shuxiu^1^; Dai, Rong^1^; Wang, Jinghang^1^; Zhu, Yaqi^1^; Zhang, Wenxiao^1^; Xu, Haoran^1^; Shen, Guofeng^1^; Shen, Huizhong^2^; Ma, Jianmin^1^; Wang, Xuejun^1^; Tao, Shu^1,2^*

1 Institute of Carbon Neutrality, Laboratory for Earth Surface Processes, College of Urban and Environmental Sciences, Peking University, Beijing, China.

2 School of Environmental Science and Engineering, Southern University of Science and Technology, Shenzhen, China

* Corresponding author: Shu Tao, Email: [taos@pku.edu.cn](mailto:taos@pku.edu.cn)

There are 2 tables and 2 figures.

**Fig. S1** Maps of annual emission densities of (A) PM_2.5_, (B) PM_>10_, (C) BC, and (D) OC in 2019.

**Fig.S2** Temporal trends of emissions of PM2.5 from various sectors with best estimation and uncertainty ranges, which are presented as 50% uncertainty intervals.

**Table S1** Emission inventory structure and major updates.

| Sector | Groups | Types | Major updates | Sector | Groups | Types | Major updates |
| --- | --- | --- | --- | --- | --- | --- | --- |
| Power generation | coal | anthracite | The impacts of ultra-low emission policies were taken into account, and point-level data were utilized for spatial interpolation. | Residential | coal | anthracite, chunk | The range of fuel types was expanded, accounting for stove transition impacts. Field-measured emission factors incorporated fugitive emissions from stoves. The spatial interpolation model for residential heating was updated. |
|  |  | coking coal |  |  |  | coking coal |  |
|  |  | bituminous |  |  |  | bituminous, chunk |  |
|  |  | lignite |  |  |  | lignite |  |
|  |  | peat |  |  |  | peat |  |
|  | oil | diesel |  |  |  | anthracite, briquettes |  |
|  |  | residue oil |  |  |  | bituminous, briquettes |  |
|  |  | crude oil |  |  | oil | diesel |  |
|  |  | LPG |  |  |  | residue oil |  |
|  | gas | natural gas |  |  |  | kerosene |  |
|  | waste | municipal waste |  |  |  | LPG |  |
|  |  | industrial waste |  |  | gas | natural gas |  |
|  | biomass | solid biomass |  |  |  | gas work gas |  |
|  |  | biogas |  |  |  | coke oven gas |  |
| Industrial combustion | coal | anthracite | Additional data on gaseous fuels were provided, along with a larger set of field-measured emission factors. |  | waste | municipal |  |
|  |  | coking coal |  |  |  | non-organized |  |
|  |  | bituminous |  |  | biomass | wood log |  |
|  |  | lignite |  |  |  | tree branch |  |
|  |  | peat |  |  |  | grass |  |
|  |  | coke |  |  |  | maize |  |
|  | oil | diesel |  |  |  | corncob |  |
|  |  | residue oil |  |  |  | wheat |  |
|  |  | crude oil |  |  |  | rice |  |
|  |  | LPG |  |  |  | sugar cane |  |
|  | gas | natural gas |  |  |  | soybean |  |
|  |  | gas flaring |  |  |  | cotton |  |
|  |  | gas works gas |  |  |  | other straws |  |
|  |  | coke oven gas |  |  |  | animal dung |  |
|  |  | blast furnace gas |  |  |  | pressed wood |  |
|  | waste | municipal waste |  |  |  | pressed straw |  |
|  |  | industrial waste |  |  |  | charcoal |  |
|  | biomass | solid biomass |  |  |  | biogas |  |
|  |  | biogas |  |  |  |  |  |
| Industrial processes | coking | beehive | Production processes for cement, iron and steel, and brickmaking were further disaggregated. The updated field-measured emission factors accounted for fugitive emissions. | Commercial | coal | anthracite, chunk | For the first time, the commercial sector was independently separated out. |
|  |  | mechanical |  |  |  | coking coal |  |
|  | iron & steel | sintering |  |  |  | bituminous, chunk |  |
|  |  | ore pellet |  |  |  | lignite |  |
|  |  | pig iron |  |  |  | peat |  |
|  |  | electric arc |  |  |  | anthracite, briquettes |  |
|  |  | open hearth |  |  |  | bituminous, briquettes |  |
|  |  | O_2_ blown |  |  | oil | diesel |  |
|  |  | hot rolling |  |  |  | residue oil |  |
|  |  | ferroalloy |  |  |  | kerosene |  |
|  | petrol | ammonia |  |  |  | LPG |  |
|  | chemical | fertilizer |  |  | gas | natural gas |  |
|  |  | catalytic cracking |  |  |  | gas work gas |  |
|  | non-ferrous metals | alumina |  |  |  | coke oven gas |  |
|  |  | aluminum |  |  | waste | municipal |  |
|  |  | lead |  |  |  | non-organized |  |
|  |  | magnesium |  |  | biomass | wood log |  |
|  |  | zinc |  |  |  | pressed wood |  |
|  |  | nickel |  |  |  | pressed straw |  |
|  |  | copper |  |  |  | charcoal |  |
|  | non-metal | lime |  |  |  | biogas |  |
|  |  | brick |  |  |  |  |  |
|  |  | cement crushing |  |  |  |  |  |
|  |  | cement hydraulic |  |  |  |  |  |
|  |  | cement griding |  |  |  |  |  |
|  |  | glass |  |  |  |  |  |
|  | mining | natural gas |  |  |  |  |  |

Table S1 Emission inventory structure and major updates (continued).

| Sector | Groups | Types | Major updates |
| --- | --- | --- | --- |
| Transportation  （only exhaust emission） | coal | rail, bituminous | Considerations were given to the effects of China's on-road vehicle emission standards, complemented by further granularity incorporated for inland waterway shipping and aviation emission sources. |
|  | oil | rail, diesel |  |
|  |  | passenger vehicles, diesel |  |
|  |  | other vehicles, diesel |  |
|  |  | small fishing boats inland, diesel |  |
|  |  | small fishing boats, ocean, diesel |  |
|  |  | other vessels, inland, diesel |  |
|  |  | other vessels, ocean, diesel |  |
|  |  | other vessels, ocean, residue oil |  |
|  |  | small fishing boats, ocean, residue oil |  |
|  |  | motorcycles/tricycles, gasoline |  |
|  |  | passenger vehicles, gasoline |  |
|  |  | other vehicles, gasoline |  |
|  |  | domestic aviation, gasoline |  |
|  |  | domestic aviation, gasoline jet fuel |  |
|  |  | domestic aviation, kerosene jet fuel |  |
|  |  | international aviation, kerosene jet fuel |  |
|  |  | other vehicles, LPG |  |
|  | gas | vehicles, natural gas |  |
|  |  | vessels, ocean, LNG |  |
|  |  | pipeline, natural gas |  |
|  | biomass | vehicles, biogas |  |
|  |  | vehicles, liquid biofuel |  |
| Agriculture | coal | bituminous | Agricultural coal use data were supplemented and updated based on yearbook information. |
|  | oil | off-road vehicles, diesel |  |
|  |  | off-road vehicles, residue oil |  |
|  | gas | natural gas |  |
|  | biomass | deforestation |  |
|  |  | crop residue, open-fire |  |
| Natural |  | peat fire |  |
|  |  | boreal forest fire |  |
|  |  | temperate forest fire |  |
|  |  | grassland fire |  |

Table S2 List of procedures for inventory compilation and parameters used for uncertainty analysis.

| **Category** | **Sector** | **Key references** | **Procedure of inventory compilation** | **Uncertainties analysis** |
| --- | --- | --- | --- | --- |
| Activity | Power generation | [1] | - Data were collected from various databases and doubled checked for temporal continuity and detect anomalies; - Production of beehive cokes were based total production from government and detected stoves using remote sensing images; - Missing data were estimated based on data for similar sources or regions using various proxies including GDP, population, and income. | - Uncertainty was addressed by using Monte Carlo simulation, for which activity data was assumed uniformed distributed with a coefficient of variation of 5%, except 10% for residential biomass. |
|  | Ind. combustion | [1,2] |  |  |
|  | Residential | [1,3–10] |  |  |
|  | Commercial | [1,5] |  |  |
|  | Transportation | [1,11,12] |  |  |
|  | Ind. processes | [11,13–18] |  |  |
|  | Agriculture | [1,19] |  |  |
|  | Natural | [19] |  |  |
| Size distribution | Power generation | [20–26] | - Size fractions of PMs were derived based on published literature, open-access or commercial databases, and technical manuals for various industrial processes; - Data gaps were filled based on data available for similar sources. | - In Monte Carlo simulation for uncertainty, uniform distribution was assumed with a coefficient of variation of 20%. |
|  | Ind. ombustion | [20,22,27,28] |  |  |
|  | Residential | [20,22,27–30] |  |  |
|  | Commercial | [27,31–34] |  |  |
|  | Transportation | [25,27,35–37] |  |  |
|  | Ind. processes | [22,25,37–40] |  |  |
|  | Agriculture | [20,22,27,37,41] |  |  |
|  | Natural | [41,42] |  |  |
| EFs | Power generation | A total of 375 references were collected for the three size fractions, and 295 references were collected for BC and OC. | - Reported EF data from several hundred literature including papers, reports, and database were collected; - The dataset was cleaned up by comparing similar data from various sources and checking the methods used in the literature; - Data gap for residential sources in developing countries were filled by over 2,000 on-site measurements conducted in China; - Data gaps for EFs for PM_10_ and TSP will filled based on reported ratios of PM_2.5_/PM_10_ and PM_2.5_/TSP. | - With large sample numbers, coefficient of variation used in Monte Carlo simulation were derived directly from the database. |
|  | Ind. combustion |  |  |  |
|  | Residential |  |  |  |
|  | Commercial |  |  |  |
|  | Transportation |  |  |  |
|  | Ind. processes |  |  |  |
|  | Agriculture |  |  |  |
|  | Natural |  |  |  |
| Improved facilities and end-of-pipe abatement | Power generation | [21,26,43–47] | - Most data were from various year books and other literature - to fill data gap of rural residents in developing countries, two filed surveys were conducted in China covering more than 90,000 households. - to model temporal trends for power plants, industrial combustion and processes using S-curves based on literature data available. - to estimate mitigation rates based on emission standard implementation and vehicle ownership statistics. | - For Monte Carlo simulation, uniform distribution was assumed with a coefficient of variation of 5%. |
|  | Ind. combustion | [42,46,48,49] |  |  |
|  | Residential | [50–54] |  |  |
|  | Commercial | [3,50,52] |  |  |
|  | Transportation | [55–59] |  |  |
|  | Ind. processes | [25,60–66] |  |  |
|  | Agriculture |  |  |  |
|  | Natural |  |  |  |
| Spatial disaggregation | Power generation | [67] | - Detailed data for residential energy consumption in China were from two field surveys covering more than 90,000 households. For other countries, population was used as a proxy and a heating degree day model was used for heating energy; - Official and commercial database with locations were used for industrial sectors. CO_2_ emissions were also used as a proxy; Locations of beehive coke overs were from remote sensing images; - Whenever available, sub-national data were collected for large countries before detailed spatial disaggregation based on various proxies were applied. | - Unable to be addressed at this stage |
|  | Ind. combustion | [5,68] |  |  |
|  | Residential | [69] |  |  |
|  | Commercial | [5,68] |  |  |
|  | Transportation | [70] |  |  |
|  | Ind. processes | [71,72] |  |  |
|  | Agriculture | [5,68] |  |  |
|  | Natural | [19] |  |  |

**SI References**

1. International Energy Agency (IEA). IEA World Energy Statistics and Balances; http://www.oecd-ilibrary.org/statistics (accessed June 20, 2023).

2. The Work Bank. *Global Gas Flaring Tracker Report*; World Bank Publications, Washington D. C., USA, 2021. <https://www.worldbank.org/en/topic/extractiveindustries/publication/2023-global-gas-flaring-tracker-report>

3. Shen G, Xiong R, Tian Y *et al.* Substantial transition to clean household energy mix in rural China. *Natl Sci Rev* 2022;**9**:nwac050.

4. Cogut A. Open Burning of Waste: A Global Health Disaster; R20 Regions of Climate Action. 2016.

5. Department of Energy Statistics, and National Bureau of Statistics of People’s Republic of China. *China Energy Statistical Yearbook 1986-2020*; China Statistics Press: Beijing, China, 1986-2020.

6. Hao W. Study on emission characteristics of air pollutants from open burning of municipal solid waste. 2019.

7. Tao S, Ru MY, Du W *et al.* Quantifying the rural residential energy transition in China from 1992 to 2012 through a representative national survey. *Nat Energy* 2018;**3**:567–73.

8. Wiedinmyer C, Yokelson RJ, Gullett BK. Global Emissions of Trace Gases, Particulate Matter, and Hazardous Air Pollutants from Open Burning of Domestic Waste. *Environ Sci Technol* 2014;**48**:9523–30.

9. Xu J, Liu S, Jiang Y *et al.* Policy analysis and implementation of biomass molding fuels industry in China. *Adv New Renew Energy* 2015;**3**:477–84.

10. Yevich R, Logan JA. An assessment of biofuel use and burning of agricultural waste in the developing world. *Glob Biogeochem Cycles* 2003;**17**.

11. International Institute for Applied Systems Analysis (IIASA). *The Greenhouse Gas - Air Pollution Interaction and Synergies (GAINS) Model*; <https://gains.iiasa.ac.at> (accessed June 20, 2023).

12. Ministry of Agriculture and Rural Affairs of the People’s Republic of China. *China Fishery Statistical Yearbook, 2006-2020*; China Agriculture Press: Beijing, China, 2006-2020.

13. Xu Y, Shen H, Yun X *et al.* Health effects of banning beehive coke ovens and implementation of the ban in China. *Proc Natl Acad Sci USA* 2018;**115**:2693–8.

14. World Steel Association (WSA). *Steel Statistic Yearbooks*; <https://worldsteel.org> (accessed June 20, 2023).

15.United States Geological Survey (USGS). *Commodity Statistics and Information*; <https://www.usgs.gov/centers/national-minerals-information-center/commodity-statistics-and-information> (accessed June 20, 2023).

16. Nation Master. <https://www.nationmaster.com/>. Accessed 22 June, 2023.

17. United Nations Statistics Division (UNSD). *Industrial Commodity Statistics Database*; <https://data.un.org> (accessed June 20, 2023).

18. China Building Materials Federation. *Almanac of China Building Materials Industry, 1981-2020*; Almanac of China Building Materials Industry Press: Beijing, China, 1982-2020.

19. van der Werf GR, Randerson JT, Giglio L *et al.* Global fire emissions estimates during 1997–2016. *Earth Syst Sci Data* 2017;**9**:697–720.

20. U.S. Environmental Protection Agency. *Air Emissions Factors and Quantification*;https://www.epa.gov/air-emissions-factors-and-quantification(25 September 2020, date last accessed).

21. Wu B, Tian H, Hao Y *et al.* Refined assessment of size-fractioned particulate matter (PM2.5/PM10/PMtotal) emissions from coal-fired power plants in China. *Sci Total Environ* 2020;**706**:135735.

22. Klimont Z, Kupiainen K, Heyes C *et al.* Global anthropogenic emissions of particulate matter including black carbon. *Atmos Chem Phys* 2017;**17**:8681–723.

23. Bond TC, Streets DG, Yarber KF *et al.* A technology-based global inventory of black and organic carbon emissions from combustion. *J Geophys Res-Atmospheres* 2004;**109**:D14203.

24. Zhao Y, Wang S, Nielsen CP *et al.* Establishment of a database of emission factors for atmospheric pollutants from Chinese coal-fired power plants. *Atmos Environ* 2010;**44**:1515–23.

25. Kurokawa J, Ohara T. Long-term historical trends in air pollutant emissions in Asia: Regional Emission inventory in ASia (REAS) version 3. *Atmos Chem Phys* 2020;**20**:12761–93.

26. Liu F, Zhang Q, Tong D *et al.* High-resolution inventory of technologies, activities, and emissions of coal-fired power plants in China from 1990 to 2010. *Atmos Chem Phys* 2015;**15**:13299–317.

27. European Environment Agency. *EMEP/EEA Air Pollutant Emission Inventory Guidebook 2019: Technical Guidance to Prepare National Emission Inventories.* LU: Publications Office, 2019.

28. Lei Y, Zhang Q, He KB *et al.* Primary anthropogenic aerosol emission trends for China, 1990–2005. *Atmos Chem Phys* 2011;**11**:931–54.

29. Shen H, Luo Z, Xiong R *et al.* A critical review of pollutant emission factors from fuel combustion in home stoves. *Environ Int* 2021;**157**:106841.

30. Chen YJ, Sheng GY, Bi XH *et al.* Emission factors for carbonaceous particles and polycyclic aromatic hydrocarbons from residential coal combustion in China. *Environ Sci Technol* 2005;**39**:1861–7.

31. Chen Y, Tian C, Feng Y *et al.* Measurements of emission factors of PM2.5, OC, EC, and BC for household stoves of coal combustion in China. *Atmos Environ* 2015;**109**:190–6.

32. Huang Y, Du W, Chen Y *et al.* Household air pollution and personal inhalation exposure to particles (TSP/PM2.5/PM1.0/PM0.25) in rural Shanxi, North China. *Environ Pollut* 2017;**231**:635–43.

33. Liu X, Guo C, Wu Y *et al.* Evaluating cost and benefit of air pollution control policies in China: a systematic review. *J Environ Sci* 2022.

34. Krugly E, Martuzevicius D, Puida E *et al.* Characterization of Gaseous- and Particle-Phase Emissions from the Combustion of Biomass-Residue-Derived Fuels in a Small Residential Boiler. *Energy & Fuels* 2014;**28**:5057–66.

35. Yan F, Winijkul E, Jung S *et al.* Global emission projections of particulate matter (PM): I. Exhaust emissions from on-road vehicles. *Atmos Environ* 2011;**45**:4830–44.

36. Franco V, Kousoulidou M, Muntean M *et al.* Road vehicle emission factors development: A review. *Atmos Environ* 2013;**70**:84–97.

37. Lükewille A, Bertok I, Amann M et al. A Framework to Estimate the Potential and Costs for the Control of Fine Particulate Emissions in Europe. Laxenburg: International Institute for Applied Systems Analysis. Report No.:IR-01-023.

38. Klimont Z, Cofala J, Bertok I et al. Modelling Particulate Emissions in Europe. Laxenburg: International Institute for Applied Systems Analysis. Report No.:IR-02-076.pdf.

39. Lei Y, Zhang Q, He KB *et al.* Primary anthropogenic aerosol emission trends for China, 1990–2005. *Atmos Chem Phys* 2011;**11**:931–54.

40. Liu J, Tong D, Zheng Y *et al.* Carbon and air pollutant emissions from China’s cement industry 1990–2015: trends, evolution of technologies, and drivers. *Atmos Chem Phys* 2021;**21**:1627–47.

41. Dennis A, Fraser M, Anderson S *et al.* Air pollutant emissions associated with forest, grassland, and agricultural burning in Texas. *Atmos Environ* 2002;**36**:3779–92.

42. Huang, Shen H, Chen H *et al.* Quantification of Global Primary Emissions of PM2.5, PM10, and TSP from Combustion and Industrial Process Sources. *Environ Sci Technol* 2014;**48**:13834–43.

43. Xu H, Zhang W, Ren Y *et al.* Role of primary drivers leading to emission reduction of major air pollutants and CO2 from global power plants. *Environ Int* 2024;**190**.

44. Zeng X, Kong S, Zhang Q *et al.* OC Source profiles and emission factors of organic and inorganic species in fine particles emitted from the ultra-low emission power plant and typical industries. *Sci Total Environ* 2021;**789**:147966.

45. Chen X, Liu Q, Yuan C *et al.* Emission characteristics of fine particulate matter from ultra-low emission power plants. *Environ Pollut* 2019;**255**:113157.

46. Nihalani SA, Mishra Y, Juremalani J. Emission Control Technologies for Thermal Power Plants. *International Conference on Recent Advances in Materials, Mechanical and Civil Engineering*. Vol 330. 2018, 012122.

47. State Council of China. GB 13223-1996 Emission standards for air pollutants in thermal power plants [in Chinese]. 1996.

48. Zhang J, Shen H, Chen Y *et al.* Iron and Steel Industry Emissions: A Global Analysis of Trends and Drivers. *Environ Sci Technol* 2023;**57**:16477–88.

49. Shen H, Huang Y, Wang R *et al.* Global Atmospheric Emissions of Polycyclic Aromatic Hydrocarbons from 1960 to 2008 and Future Predictions. *Environ Sci Technol* 2013;**47**:6415–24.

50. Meng W, Shen G, Shen H *et al.* Synergistic Health Benefits of Household Stove Upgrading and Energy Switching in Rural China. *Environ Sci Technol* 2021.

51. Shen G, Ru M, Du W *et al.* Impacts of air pollutants from rural Chinese households under the rapid residential energy transition. *Nat Commun* 2019;**10**:3405.

52. Zhang W, Yun X, Meng W *et al.* Urban residential energy switching in China between 1980 and 2014 prevents 2.2 million premature deaths. *One Earth* 2021;**4**:1602–13.

53. Meng W, Zhong Q, Chen Y *et al.* Energy and air pollution benefits of household fuel policies in northern China. *Proc Natl Acad Sci USA* 2019;**116**:16773–80.

54. Luo Z, Shen G, Men Y *et al.* Reduced inequality in ambient and household PM2.5 exposure in China. *Environ Int* 2022;**170**:107599.

55. Ministry of Ecology and Environment of the People’s Republic of China; General Administration of Quality Supervision, Inspection and Quarantine of the People’s Republic of China. *Limits and Measurement Methods for Emissions from Light-duty Vehicles (China 5) (GB 18352.6-2013)*; China Environment Publishing Group: Beijing, China, 2013. <https://www.mee.gov.cn/ywgz/fgbz/bz/bzwb/dqhjbh/dqydywrwpfbz/201309/t20130917_260352.shtml> (accessed June 21, 2023).

56. Ministry of Ecology and Environment of the People’s Republic of China; General Administration of Quality Supervision, Inspection and Quarantine of the People’s Republic of China. *Limits and Measurement Methods for Emissions from Light-duty Vehicles (China 6) (GB 18352.6-2016)*; China Environment Publishing Group: Beijing, China, 2016. <https://www.mee.gov.cn/ywgz/fgbz/bz/bzwb/dqhjbh/dqydywrwpfbz/201612/t20161223_369476.shtml> (accessed June 21, 2023).

57. Ministry of Ecology and Environment of the People’s Republic of China; General Administration of Quality Supervision, Inspection and Quarantine of the People’s Republic of China. *Limits and Measurement Methods for Emissions from Light-duty Vehicles (Ⅰ) (GB 18352.1-2001)*; China Environment Publishing Group: Beijing, China, 2001. <https://www.mee.gov.cn/ywgz/fgbz/bz/bzwb/dqhjbh/dqydywrwpfbz/200104/t20010416_67420.shtml> (accessed June 21, 2023).

58. Ministry of Ecology and Environment of the People’s Republic of China; General Administration of Quality Supervision, Inspection and Quarantine of the People’s Republic of China. *Limits and Measurement Methods for Emissions from Light-duty Vehicles (Ⅲ, Ⅳ) (GB 18352.3-2005)*; China Environment Publishing Group: Beijing, China, 2005. <https://www.mee.gov.cn/ywgz/fgbz/bz/bzwb/dqhjbh/dqydywrwpfbz/200707/t20070701_66145.shtml> (accessed June 21, 2023).

59. Ministry of Ecology and Environment of the People’s Republic of China; State Administration for Market Regulation. *Limits and Measurement Methods for Emissions from Diesel Fueled Heavy-duty Vehicles (China VI) (GB 17691-2018)*; 2018. <https://www.mee.gov.cn/ywgz/fgbz/bz/bzwb/dqhjbh/dqydywrwpfbz/201807/t20180703_445995.shtml> (accessed June 21, 2023).

60. Hua S, Tian H, Wang K *et al.* Atmospheric emission inventory of hazardous air pollutants from China’s cement plants: Temporal trends, spatial variation characteristics and scenario projections. *Atmos Environ* 2016;**128**:1–9.

61. Lei Y, Zhang Q, Nielsen C *et al.* An inventory of primary air pollutants and CO2 emissions from cement production in China, 1990–2020. *Atmos Environ* 2011;**45**:147–54.

62. Liu J, Tong D, Zheng Y *et al.* Carbon and air pollutant emissions from China’s cement industry 1990–2015: trends, evolution of technologies, and drivers. *Atmos Chem Phys* 2021;**21**:1627–47.

63. Li Z, Hu Y, Chen L *et al.* Emission Factors of NOx, SO2, and PM for Bathing, Heating, Power Generation, Coking, and Cement Industries in Shanxi, China: Based on Field Measurement. *Aerosol Air Qual Res* 2018;**18**:3115–26.

64. Wang K, Tian H, Hua S *et al.* A comprehensive emission inventory of multiple air pollutants from iron and steel industry in China: Temporal trends and spatial variation characteristics. *Sci Total Environ* 2016;**559**:7–14.

65. Wang X, Lei Y, Yan L *et al.* A unit-based emission inventory of SO2, NOx and PM for the Chinese iron and steel industry from 2010 to 2015. *Sci Total Environ* 2019;**676**:18–30.

66. Hu Y, Li Z, Wang Y *et al.* Emission Factors of NOx, SO2, PM and VOCs in Pharmaceuticals, Brick and Food Industries in Shanxi, China. *Aerosol Air Qual Res* 2019;**19**:1785–97.

67. Byers L, Friedrich J, Hennig R *et al.* A Global Database of Power Plants. Washington DC, World Resources Institute. 2019. <https://www.wri.org/research/global-database-power-plants>. Accessed July 12, 2023.

68. National Bureau of Statistics and National Energy Administration. CHINA INDUSTRY STATISTICAL YEARBOOK(2021).https://data.cnki.net/yearBook/single?id=N2024050101(24 May 2022, date last accessed).

69. Chen H, Huang Y, Shen H *et al.* Modeling temporal variations in global residential energy consumption and pollutant emissions. *Appl Energ* 2016;**184**:820–9.

70. European Commission, Joint Research Centre (JRC)/Netherlands Environmental Assessment Agency (PBL). *Emissions Database for Global Atmospheric Research, release version 6.1 (EDGAR_v6.1)*; <https://edgar.jrc.ec.europa.eu/dataset_ap61#p1> (accessed June 24, 2023).

71. Lei T, Guan D, Shan Y *et al.* Adaptive CO2 emissions mitigation strategies of global oil refineries in all age groups. *One Earth* 2021;**4**:1114–26.

72. Carbon Emission Accounts & Datasets (CEADs). <https://www.ceads.net.cn/data/> (accessed June 25, 2023).
